# Hidden in the Scar: A Rare Case of Vulvar Endometriosis Mimicking Bartholin′s Cyst at an Episiotomy Site

**DOI:** 10.1155/crog/7202665

**Published:** 2026-03-09

**Authors:** Swati Kumari, Anna Zaradna, Valmiki Vijay Seeraj

**Affiliations:** ^1^ Department of Obstetrics and Gynecology, BronxCare Health System, Bronx, New York, USA, bronxcare.org

**Keywords:** Bartholin′s cyst mimic, episiotomy, scar endometriosis, vulvar endometriosis

## Abstract

**Background:**

Vulvar endometriosis is an exceptionally rare manifestation of extrapelvic endometriosis, particularly when located at the site of a prior episiotomy. Often misdiagnosed as more common vulvar pathologies, these lesions may present with cyclical pain and swelling, mimicking Bartholin′s gland cysts or infected epidermal inclusion cysts.

**Case:**

We report the case of a 25‐year‐old gravida 3 para 2 woman with long‐standing dysmenorrhea, dyspareunia, and a right vulvar mass that fluctuated with her menstrual cycle. Initially presumed to be a Bartholin′s cyst, the lesion failed to respond to antibiotics and sitz baths. MRI revealed a complex vaginal wall cyst without classic signs of pelvic endometriosis. Examination under anesthesia and aspiration of chocolate‐colored fluid raised suspicion for an endometriotic lesion. Surgical excision confirmed endometrial glands and stroma with hemosiderin‐laden macrophages—consistent with vulvar endometriosis at the site of a right mediolateral episiotomy scar. Postoperative recovery was uneventful, and the patient experienced complete resolution of symptoms.

**Conclusion:**

This case highlights the diagnostic challenge of vulvar endometriosis in women with prior perineal trauma. Clinicians should maintain a high index of suspicion for endometriosis in cyclical vulvar masses—especially when located along episiotomy scars and unresponsive to conventional treatment. Early recognition and surgical excision can be curative and significantly improve quality of life.

## 1. Introduction

Endometriosis is a chronic, estrogen‐dependent inflammatory condition defined by the presence of endometrial glands and stroma outside the uterine cavity. It most commonly affects pelvic structures such as the ovaries, fallopian tubes, uterosacral ligaments, and pouch of Douglas. However, extrapelvic manifestations—though rare—have been documented in nearly every organ system, including the gastrointestinal tract, lungs, and cutaneous tissues. Vulvar endometriosis is a rare manifestation of extrapelvic endometriosis and may be misdiagnosed due to its nonspecific clinical presentation [1].

Scar endometriosis, particularly following obstetric or gynecologic surgeries such as cesarean sections and episiotomies, is a recognized but underreported entity. Although most scar endometriosis occurs within the abdominal wall postcesarean, its occurrence at the episiotomy site is exceedingly uncommon. Clinicians often misattribute the symptoms—such as vulvar swelling, cyclical pain, or dyspareunia—to more common conditions like Bartholin′s cysts, abscesses, or epidermal inclusion cysts [2, 3].

This case describes a young woman with cyclic vulvar swelling and pain misattributed for over a year as an infected epidermoid cyst or Bartholin′s gland cyst. Ultimately, she was found to have a rare presentation of vulvar endometriosis at the site of a prior right mediolateral (RML) episiotomy. This case underscores the importance of considering endometriosis in the differential diagnosis of recurrent or cyclic perineal masses, especially in women with prior obstetric interventions and suggestive menstrual associations. It also highlights the diagnostic and therapeutic challenges of this rare but impactful condition.

## 2. Case Summary

A 25‐year‐old gravida 3 para 2 presented to the gynecology clinic for assessment and management of a right‐sided vulvar mass noted after an emergency‐department visit for cyclical right vulvar swelling that became more prominent during menses and lasted up to 1 week afterward.

The patient has a history of irregular menses, long‐standing dysmenorrhea, and both deep and superficial dyspareunia. She has had one cesarean section followed by a vaginal birth after cesarean (VBAC) with a RML episiotomy. Surgical history includes right salpingectomy and right ovarian cystectomy for an ectopic pregnancy, and interval female sterilization after completing childbearing. She was previously diagnosed and treated for syphilis. She denied abnormal vaginal or vulvar discharge and denies abdominal or pelvic pain.

During the ED visit, gynecology was consulted for a suspected Bartholin‐gland cyst. The patient reported a painful right vulvar lump present for more than a year. It had been treated as an infected epidermoid cyst with trimethoprim–sulfamethoxazole, sitz baths, and chlorhexidine soap, yielding only partial symptomatic improvement with no change in size or tenderness during the menstrual cycle. Pelvic MRI showed a 1.3 × 1.5 × 1.6 − cm complex cyst in the right vaginal wall described as “likely Bartholin′s cyst,” dilated bilateral gonadal veins suggestive of pelvic congestion, and a small amount of free pelvic fluid; no ovarian mass or other signs of endometriosis were noted.

**Figure 1:**
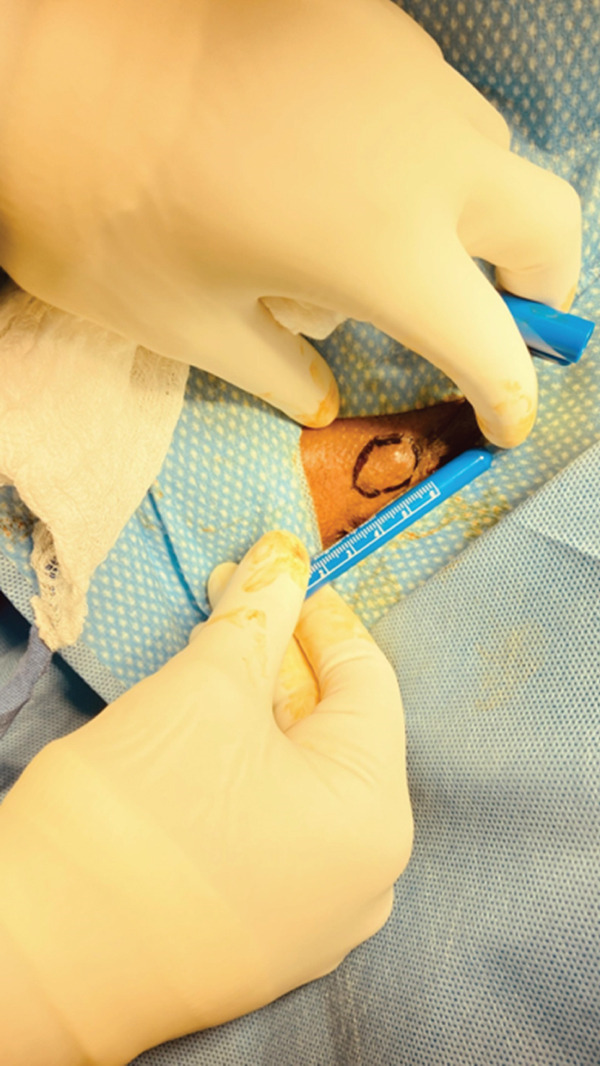
Vulvar cyst at the episiotomy scar site before incision.

At outpatient follow‐up, the mass measured 2 × 1.5 cm, was well demarcated and firm, and lay subcutaneously in the perineum at 7 o′clock to the introitus, along the scar of the previous RML episiotomy, about 1 cm distal to the fourchette (Figure 1).

Aspiration yielded a small amount of chocolate‐colored fluid, raising suspicion for an endometriotic deposit. Combined oral contraceptives were started for ovulation suppression. Surgical excision was offered, but the patient was lost to follow‐up.

Four months later, she returned and elected surgical management. After informed consent—including discussion of pain, bleeding, infection, wound dehiscence, and possible lack of symptom relief—she underwent scheduled excision in March 2025.

**Figure 2:**
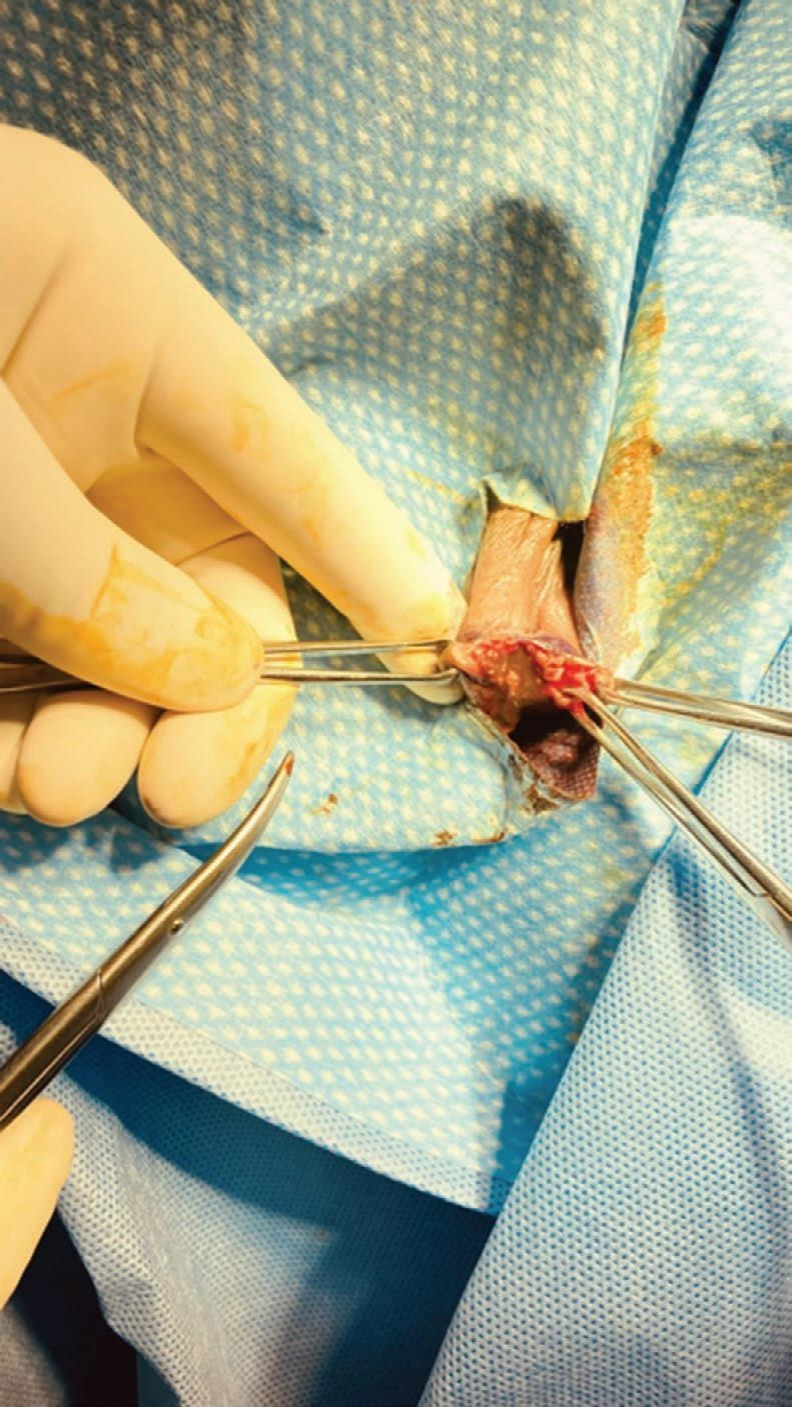
Surgical excision of the vulvar endometriotic lesion.

Under general and local anesthesia (lidocaine with epinephrine), examination under anesthesia confirmed a 2 × 1.5 − cm firm, oval, well‐demarcated mass at 7 o′clock to the introitus, along the previous episiotomy scar. Allis clamps elevated the skin, and a #15 scalpel opened the lesion. A small amount of thick black–brown (“chocolate”) fluid was expressed (Figure 2).

No discrete capsule was seen. A 1–2‐cm nodular area of fat corresponding to the lesion was sharply excised. The cavity was closed in layers with 2‐0 Vicryl to obliterate dead space; skin was approximated with running 3‐0 Monocryl subcuticular sutures. Hemostasis was adequate and estimated blood loss minimal.

### 2.1. Postoperative Course

Two weeks post‐op, the right vulvar area was healing well with no infection; a 1 × 0.5 − cm residual swelling was noted. The patient was advised to continue sitz baths and return in 3 months.

Pathology showed endometrial glands and stroma with hemosiderin‐laden macrophages, confirming vulvar endometriosis.

At the 3‐month visit, the patient reported resolution of cyclical swelling and pain. Examination showed a well‐healed scar with minimal subcutaneous irregularity. She was advised to return in 3 months for surveillance and was counseled on the risk of recurrence and options for medical or repeat surgical management.

## 3. Discussion

Endometriosis affects approximately 10% of reproductive‐age women and is characterized by the ectopic presence of endometrial glands and stroma outside the uterine cavity [1]. Although it most commonly involves the ovaries, fallopian tubes, and peritoneal surfaces, extrapelvic endometriosis constitutes a small but clinically significant subset. Cutaneous and subcutaneous endometriosis account for less than 1% of cases and most often present in surgical scars, particularly after cesarean sections or laparotomies [2, 3]. Endometriosis involving the vulva, particularly at the site of a prior episiotomy, has been infrequently reported in the literature. [4].

The pathogenesis of vulvar endometriosis remains incompletely understood, but several hypotheses have been proposed. The most widely accepted is the iatrogenic implantation theory, which suggests direct mechanical transplantation of endometrial cells during obstetric or gynecological procedures [5]. During episiotomy or vaginal delivery, endometrial cells from the uterus or lower genital tract may be introduced into the disrupted perineal tissues, where they implant and, under continued estrogen stimulation, proliferate. This mechanism is supported by the strong temporal relationship between obstetric trauma and onset of symptoms in reported cases, including the present case, where the mass developed along the scar of a prior RML episiotomy.

Alternative theories include coelomic metaplasia, in which pluripotent mesothelial cells differentiate into endometrial tissue under hormonal or immunological influences, and lymphatic or hematogenous spread, although these are less commonly invoked in isolated vulvar lesions without pelvic disease [6].

Clinically, vulvar endometriosis can be deceptive. Most patients present with a painful vulvar mass that demonstrates cyclical changes—enlarging and becoming tender around menstruation. However, this cyclical nature may not always be initially recognized, especially in patients with irregular menses or overlapping gynecologic symptoms such as dysmenorrhea or dyspareunia [7]. Misdiagnosis is common, as in this case, where the lesion was presumed to be a Bartholin′s gland cyst or infected epidermoid cyst for over a year, and even treated with antibiotics. Notably, the presence of a chocolate‐colored aspirate and the lesion′s location along a surgical scar were key diagnostic clues.

Imaging, such as pelvic MRI, can support the diagnosis. On MRI, endometriotic lesions often appear as hyperintense or complex cystic structures on T1‐weighted images with variable enhancement on T2‐weighted imaging. However, findings may be nonspecific or misattributed to more common pathologies like cysts or abscesses, as occurred in this case. In such cases, a high index of clinical suspicion is important, particularly when lesions are firm, painful, scar‐associated, and exhibit cyclical symptoms. [8].

Histopathology remains the gold standard for diagnosis. The presence of endometrial glands, stroma, and hemosiderin‐laden macrophages confirms the diagnosis. In this patient, excision yielded tissue with all three classic histologic components. In this case, no discrete capsule was identified, suggesting a potentially infiltrative lesion, which may complicate complete excision. [9].

Management of vulvar endometriosis depends on symptom severity and patient preference. First‐line therapy often includes hormonal suppression, such as combined oral contraceptives, progestins, or GnRH analogues, which reduce estrogen stimulation and may shrink lesions. Hormonal therapy may provide symptom control in some patients; however, surgical excision is often required for definitive management of localized subcutaneous lesions [10]. Complete excision, including surrounding fibrotic tissue, is essential to minimize recurrence. In this case, despite initial partial symptom control with oral contraceptives, the patient opted for surgical removal, resulting in resolution of symptoms at 3‐month post‐op.

As with other forms of endometriosis, recurrence following treatment remains a consideration. Long‐term follow‐up is advised, especially for lesions in anatomically complex or hormonally responsive locations. Patients should also be counseled on the potential for recurrent symptoms and the role of hormonal suppression postoperatively if needed. Although pelvic endometriosis was not identified on imaging in this case, it is prudent to monitor patients with extrapelvic manifestations, as concurrent or future pelvic disease may still emerge [11].

## 4. Conclusion

In summary, this case highlights a rare but important differential diagnosis for persistent or cyclical vulvar masses, particularly in women with a history of episiotomy. It underscores the importance of clinical vigilance, thorough history‐taking, and consideration of surgical scar endometriosis in women with menstrual‐associated perineal symptoms. Early recognition and definitive excision can significantly improve quality of life and avoid unnecessary treatments or delays in care.

## Author Contributions

**Swati Kumari:** conceptualization, data curation, investigation, writing – original draft, writing – review and editing. **Anna Zaradna:** data curation, writing – review and editing. **Valmiki Vijay Seeraj:** supervision, writing – review and editing.

## Funding

The authors received no external funding for this work.

## Disclosure

The manuscript was presented at the 2026 SAAOG Annual Meeting.

## Ethics Statement

Ethical approval was not required for this case report in accordance with institutional policies. Written informed consent was obtained from the patient for publication of this case and associated images.

## Conflicts of Interest

The authors declare no conflicts of interest.

## Data Availability

Data sharing is not applicable to this article as no datasets were generated or analyzed during the current study.
